# Draft Genome Sequence of *Paenibacillus* sp. Strain MY03, a Terrestrial Bacterium Capable of Degrading Multiple Marine-Derived Polysaccharides

**DOI:** 10.1128/genomeA.00678-17

**Published:** 2017-07-20

**Authors:** Huihui Liu, Yuanyuan Cheng, Jingyan Gu, Yanhui Wang, Junge Li, Fuchuan Li, Wenjun Han

**Affiliations:** a National Glycoengineering Research Center, Shandong Provincial Key Laboratory of Carbohydrate Chemistry and Glycobiology, Shandong University, Jinan, China; b Jinan Enlighten Biotechnology Co., Ltd., Jinan, China; c Department of Food Science and Engineering, Shandong Agriculture and Engineering University, Jinan, China

## Abstract

The bacterium *Paenibacillus* sp. strain MY03, isolated from the root soil of cypress, can effectively degrade marine-derived polysaccharides such as agar, alginate, and chitin. Here, we present the draft genome sequence of MY03. Putative enzymes, including 3 agarases, 1 alginate lyase, and 1 chitinase, were found.

## GENOME ANNOUNCEMENT

The genus *Paenibacillus* was first described in 1993 to accommodate a monophyletic lineage of endospore-forming bacteria belonging to the genus *Bacillus* (1, 2). *P. polymyxa* was chosen and retained as the type species (3). The number of *Paenibacillus* spp. has increased from about 50 to more than 80 since 2005. However, so far, strains of only two species—*P. agarexedens* (4) and *P. agaridevorans* (5)—have been identified as agar-degrading bacteria, and only the species of *P. alginolyticus* and *P. chondroitinus* (6) are associated with alginate degradation.

We isolated the polysaccharide-degrading bacterium *Paenibacillus* sp. strain MY03 in April 2006 from root soil samples of cypress in the forest park of Mianyang, China. This strain could efficiently degrade and utilize multiple polysaccharides, particularly marine-derived polymers, such as agar, alginate, and chitin. It has been deposited in the China General Microbiological Culture Collection Center (accession no. CGMCC 13707).

The genome of MY03 was sequenced using Illumina/Solexa MiSeq technology at the Shanghai Majorbio Bio-pharm Technology Co., Ltd. (Shanghai, China). A library with a fragment length of 380 bp was constructed, and a total of 1,918 Mb of paired-end (300-bp) reads were generated. Approximately 1,869 Mb of high-quality reads, which provided a 205-fold depth of coverage, were assembled with SOAPdenovo version 2.04 (7). Protein-coding sequences were predicted by Glimmer version 3.02 software (8) using default parameters and annotated using BLAST searches of nonredundant protein sequences from the NCBI, Swiss-Prot, TrEMBL, COG (9), and KEGG (10) databases. For gene annotation, only significant BLAST matches with an *E* value of ≤10^−5 ^were adopted. Ribosomal RNA genes were detected using Barrnap version 0.4.2 software (11), and tRNAs were detected using tRNAscan-SE version 1.3.1 (12).

The genome size of MY03 was estimated to be 9,080,284 bp from 2 scaffolds (138 contigs) with a 51.8% G+C content. A total of 7,745 coding sequences were identified, including 57 RNA genes (2 rRNAs and 53 tRNAs).

Marine algae contain diverse polysaccharides. For instance, red algae contain agars, xylans, and starch; green algae contain sulfate galactan and xylans; and brown algae contain alginate and laminarin (β-1,3-glucan) (13). Marine animals and fungi produce chitin. In the whole genome of MY03, various polysaccharidase genes related to seaweed degradation were found, including a glucoamylase (EC 3.2.1.3), a mannanase (EC 3.2.1.78), an alginate lyase (EC 4.2.2.3 and EC 4.2.2.11), 3 putative agarases (EC 3.2.1.81), 4 glucanases (EC 3.2.1.176, EC 3.2.1.39, EC 3.2.1.4, and EC 3.2.1.6), and 10 xylanases (EC 3.2.1.8 and EC 3.2.1.37). A chitinase related to marine animal degradation was found. These results suggest an amazing capability of strain MY03 to metabolize polysaccharides of marine algae and animals. Further studies regarding the functions and origins of so many marine polysaccharidases of the terrestrial bacterium MY03 will be beneficial to the exploration of novel polysaccharide-degrading enzymes and will improve the understanding of their evolutionary processes.

### Accession number(s).

This whole-genome shotgun project has been deposited at DDBJ/ENA/GenBank under the accession number MXQD00000000. The version described in this paper is the first version, MXQD01000000.

## References

[1] Ash C, Priest FG, Collins MD 1993 Molecular identification of rRNA group 3 bacilli (Ash, Farrow, Wallbanks and Collins) using a PCR probe test. Antonie Leeuwenhoek 64:253–260. doi:10.1007/BF00873085. 8085788

[2] International Journal of Systematic Bacteriology 1994 Validation of the publication of new names and new combinations previously effectively published outside the IJSB. List no. 51 Int J Syst Evol Bacteriol 44:852. 10.1099/00207713-46-2-625 8934914

[3] Tindall BJ 2000 What is the type species of the genus *Paenibacillus*? Request for an opinion. Int J Syst Evol Microbiol 50:939–940. doi:10.1099/00207713-50-2-939. 10758908

[4] Wieringa KT 1941 *Bacillus agar-exedens*, a new species, decomposing agar. Antonie Leeuwenhoek 7:121–127. doi:10.1007/BF02351762.

[5] Uetanabaro AP, Wahrenburg C, Hunger W, Pukall R, Spröer C, Stackebrandt E, de Canhos VP, Claus D, Fritze D 2003 *Paenibacillus agarexedens* sp. nov., nom. rev., and *Paenibacillus agaridevorans* sp. nov. Int J Syst Evol Microbiol 53:1051–1057. doi:10.1099/ijs.0.02420-0. 12892125

[6] Nakamura LK 1987 *Bacillus alginolyticus* sp. nov. and *Bacillus chondroitinus* sp. nov., two alginate-degrading species. Int J Syst Evol Microbiol 37:284–286. doi:10.1099/00207713-37-3-284.

[7] Li R, Li Y, Kristiansen K, Wang J 2008 SOAP: short oligonucleotide alignment program. Bioinformatics 24:713–714. doi:10.1093/bioinformatics/btn025. 18227114

[8] Delcher AL, Bratke KA, Powers EC, Salzberg SL 2007 Identifying bacterial genes and endosymbiont DNA with Glimmer. Bioinformatics 23:673–679. doi:10.1093/bioinformatics/btm009. 17237039 PMC2387122

[9] Tatusov RL, Natale DA, Garkavtsev IV, Tatusova TA, Shankavaram UT, Rao BS, Kiryutin B, Galperin MY, Fedorova ND, Koonin EV 2001 The COG database: new developments in phylogenetic classification of proteins from complete genomes. Nucleic Acids Res 29:22–28. doi:10.1093/nar/29.1.22. 11125040 PMC29819

[10] Kanehisa M, Goto S, Kawashima S, Okuno Y, Hattori M 2004 The KEGG resource for deciphering the genome. Nucleic Acids Res 32:D277–D280. doi:10.1093/nar/gkh063. 14681412 PMC308797

[11] Seemann T 2014 Prokka: rapid prokaryotic genome annotation. Bioinformatics 30:2068–2069. doi:10.1093/bioinformatics/btu153. 24642063

[12] Lowe TM, Eddy SR 1997 TRNAscan-SE: a program for improved detection of transfer RNA genes in genomic sequence. Nucleic Acids Res 25:955–964. 9023104 10.1093/nar/25.5.955 PMC146525

[13] Kraan S 2012 Algal polysaccharides, novel applications and outlook, Chapter 22. *In* Carbohydrates—comprehensive studies on glycobiology and glycobiotechnology. IN TECH, Rijeka, Croatia. doi:10.5772/51572.

